# The Y227N mutation affects bestrophin-1 protein stability and impairs sperm function in a mouse model of Best vitelliform macular dystrophy

**DOI:** 10.1242/bio.041335

**Published:** 2019-06-14

**Authors:** Andrea Milenkovic, Denise Schmied, Naoyuki Tanimoto, Mathias W. Seeliger, Janet R. Sparrow, Bernhard H. F. Weber

**Affiliations:** 1 Institute of Human Genetics, University of Regensburg, 93053 Regensburg, Germany; 2 Division of Ocular Neurodegeneration, Centre for Ophthalmology, Institute for Ophthalmic Research, 72076 Tübingen, Germany; 3 Department of Ophthalmology, University of Kiel, 24105 Kiel, Germany; 4 Department of Ophthalmology, Harkness Eye Institute, Columbia University Medical Center, 10032 New York, USA

**Keywords:** Bestrophin-1, BEST1, Vitelliform macular dystrophy, Protein stability, Sperm motility, Best1^Y227N^ knock-in mouse

## Abstract

Human bestrophin-1 (BEST1) is an integral membrane protein known to function as a Ca^2+^-activated and volume-regulated chloride channel. The majority of disease-associated mutations in *BEST1* constitute missense mutations and were shown *in vitro* to lead to a reduction in mutant protein half-life causing Best disease (BD), a rare autosomal dominant macular dystrophy. To further delineate BEST1-associated pathology *in vivo* and to provide an animal model useful to explore experimental treatment efficacies, we have generated a knock-in mouse line (Best1^Y227N^). Heterozygous and homozygous mutants revealed no significant ocular abnormalities up to 2 years of age. In contrast, knock-in animals demonstrated a severe phenotype in the male reproductive tract. In heterozygous Best1^Y227N^ males, Best1 protein was significantly reduced in testis and almost absent in homozygous mutant mice, although mRNA transcription of wild-type and knock-in allele is present and similar in quantity. Degradation of mutant Best1 protein in testis was associated with adverse effects on sperm motility and the capability to fertilize eggs. Based on these results, we conclude that mice carrying the *Best1* Y227N mutation reveal a reproducible pathologic phenotype and thus provide a valuable *in vivo* tool to evaluate efficacy of drug therapies aimed at restoring Best1 protein stability and function.

## Abstract (precis)

**Summary:** Mice carrying the human Y227N mutation in the endogenous *Best1* gene reveal a pathologic phenotype in testis providing an *in vivo* tool to evaluate therapeutic efficacies aimed at restoring protein function.

## INTRODUCTION

Bestrophin-1 (*BEST1*) was initially identified via positional cloning of the gene causing the autosomal dominant Best vitelliform macular dystrophy, also known as Best disease (BD) (Marquardt et al., 1998; Petrukhin et al., 1998). Since then, over 250 distinct *BEST1* mutations have been linked to BD, and later to a lesser extent also to three other distinct retinal pathologies (https://databases.lovd.nl/shared/genes/BEST1, https://www.ncbi.nlm.nih.gov/clinvar), namely the autosomal dominant adult-onset vitelliform macular dystrophy (AVMD) (Krämer et al., 2000), the autosomal dominant vitreoretinal choroidopathy (ADVIRC) (Yardley et al., 2004) and the autosomal recessive bestrophinopathy (ARB) (Burgess et al., 2008). Key features of BEST1-related pathology include subretinal egg yolk-like (vitelliform) lesions (Mohler and Fine, 1981), fluid- and debris-filled retinal detachments (Mohler and Fine, 1981) and a reduction in the electro-oculogram (EOG) light peak (Cross and Bard, 1974).

Structural and functional analysis of BEST1 established the protein as a Ca^2+^-activated and volume-regulated chloride channel (Sun et al., 2002; Hartzell et al., 2008; Xiao et al., 2008; Milenkovic et al., 2015) by forming a homo-pentameric protein complex (Kane Dickson et al., 2014; Yang et al., 2014). To further investigate disease pathology, several *in vitro* and *in vivo* disease models were generated. First, we and others showed that MDCKII BEST1-transfected cell culture models are well suited to demonstrate mis-localization (Johnson et al., 2014, 2013; Milenkovic et al., 2011) and reduced protein stability (Uggenti et al., 2016; Milenkovic et al., 2018) of autosomal dominant as well as autosomal recessive *BEST1* mutations. These observations were further supported by studies in BD patient-derived human induced pluripotent stem cell (hiPSC) retinal pigment epithelium (RPE) (Milenkovic et al., 2015; Singh et al., 2015; Marmorstein et al., 2018). BD hiPSC-RPE cells displayed reduced protein expression (Milenkovic et al., 2015; Marmorstein et al., 2018) that was correlated with a diminished chloride conductance (Moshfegh et al., 2016) and delayed digestion of photoreceptor outer segments (POS) (Singh et al., 2015; Marmorstein et al., 2018). As such, MDCKII and hiPSC-RPE cells offer valuable *in vitro* tools to search for chemical compounds that modulate protein stability and possibly the degradative processes of mutant BEST1. Furthermore, spontaneous autosomal recessive mutations in the *Best1* gene of dog breeds were reported providing a canine model of ARB (Guziewicz et al., 2007; Zangerl et al., 2010). Recently, for the canine ARB breed it was demonstrated that severity and progression can be attenuated by pharmacological intervention (Singh et al., 2015) or by adeno-associated virus 2 (AAV2)-mediated *BEST1* gene augmentation (Guziewicz et al., 2018). A BD-associated *Best1* knock-in mouse line carrying the autosomal dominant W93C BEST1 mutation (Best1^W93C^) displayed key features of the BD phenotype in the mouse eye (Zhang et al., 2010). While this phenotype was associated with potential abnormalities in calcium homeostasis, a direct link of these findings with the mutant Best1 protein and the ocular phenotype has not been established so far.

Here, we sought to establish a novel *Best1* knock-in by introducing the recurrent human BD mutation Y227N into the mouse germline. This mouse model can be used to further delineate a BEST1-associated phenotype and to monitor potential treatment effects. We analyzed normal and mutant murine Best1 (mBest1) expression and addressed further functional aspects, specifically in two murine tissues, namely the RPE/retina complex and the testis, the latter as the site of highest endogenous Best1 expression in the mouse (Krämer et al., 2004; Milenkovic et al., 2015). Of note, our analysis of mutant knock-in mice (Best1^Y227N^) revealed no sign of functional pathology in the eye in heterozygous nor in homozygous animals. In contrast, we observed severely reduced Best1 protein expression in the testis from homozygous Best1^Y227N^ mice characterized by high levels of mono-ubiquitinated Best1 protein. The findings in Best1^Y227N^ mouse testis were directly associated with a decrease in sperm motility and the capability to fertilize eggs. This strong phenotype offers an excellent *in vivo* model to test drug efficacy of BEST1-associated pathology.

## RESULTS

### Generation of knock-in mice harboring the mBest1-Y227N mutation

**Fig. 1.**
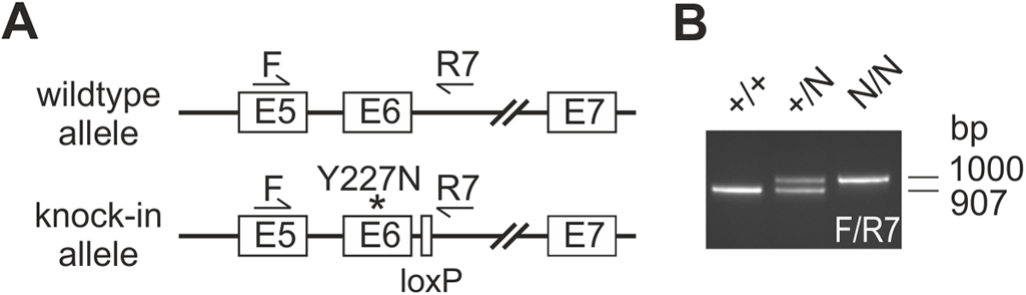
**Construction of a Best1 knock-in targeting vector.** (A) Schematic representation of the targeting strategy to generate ES cells which harbor the Y227N mutation in exon 6 of the *mBest1* gene flanked by a loxP site. (B) Mice were genotyped by PCR amplification of DNA extracted from mouse tail resulting in a 907 bp fragment in +/+ and an additional 1.000 bp fragment in +/N mice. The 1.000 bp fragment containing the loxP site is indicative of the correct targeting of the *Best1* allele.

Known disease-causing mutations in the *BEST1* gene are clustered in the N-terminal half of the protein, with no striking preference for a particular codon or protein motif. The T to A transversion that encodes a tyrosine-to-asparagine change at codon 227 (Y227N) was identified in the affected members of an Iowa family of Dutch ancestry and also in a Canadian four-generation family from our patient cohort (Courtney et al., 2014), all affected individuals presenting with clinical features typical of BD (Petrukhin et al., 1998; Mullins et al., 2005). The Y227N mutation was introduced into the endogenous *mBest1* gene via homologous recombination (Fig. 1A). After removal of the neomycin cassette via Cre recombinase, PCR and Sanger sequencing was performed to confirm correct targeting into the mouse genome (Fig. 1B). Prior to phenotypic analysis, the mutant allele was crossed for more than ten generations onto a C57BL/6J (B6/J) and the outbred CD-1 genetic background, respectively. Heterozygous Best1^Y227N^ mice (+/N) gave rise to apparently healthy offspring at the expected Mendelian frequency (data not shown).

### Expression profiling of mBest1 in the mouse eye

**Fig. 2.**
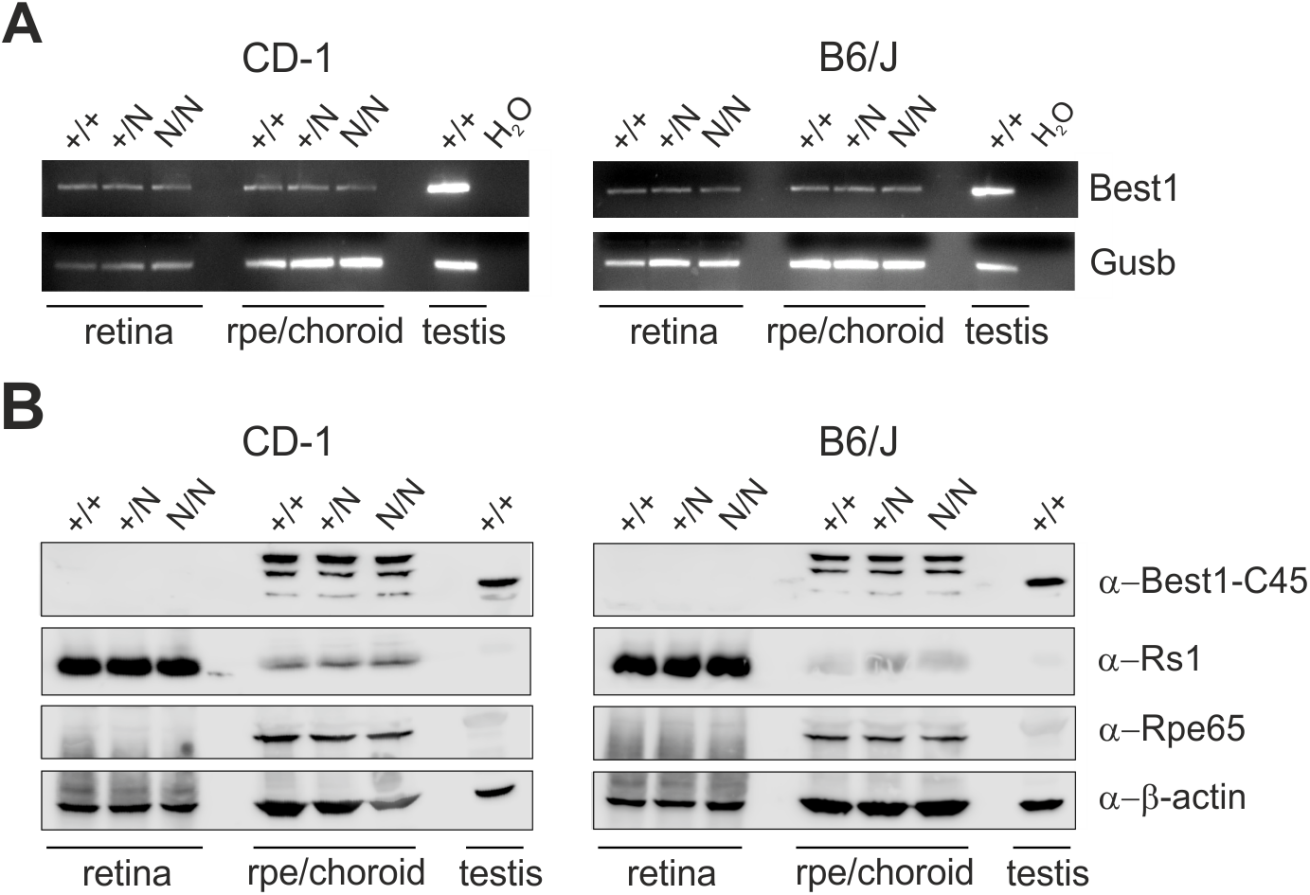
**RNA and protein expression analysis of Best1 in various tissues of Best1^Y227N^ mice.** (A) RNA expression of *Best1* in mouse tissues of retina, RPE/choroid and testis from CD-1 (left graph) and B6/J (right graph) +/+, +/N, and N/N by RT-PCR. *Gusb* (glucuronidase, beta) served as a control for RNA integrity. (B) Western blot analysis of whole cell lysates of indicated mouse tissues from CD-1 (left graph) and B6/J (right graph) +/+, +/N and N/N mice using polyclonal antibody α-Best1-C45 detecting the C-terminus of mBest1 (Milenkovic et al., 2015). Co-staining was performed with cell-specific markers for retina (α-Rs1) and RPE (α-Rpe65) on the same blot. Anti-beta actin served as control for equal protein loading.

Murine Best1 expression was found in a variety of tissues including the RPE/retina, although highest expression was eminent in the murine testis (Krämer et al., 2004; Milenkovic et al., 2015). To determine whether the introduction of the Y227N mutation affects mRNA or protein expression, we performed reverse transcription (RT)-PCR and western blot analysis from retina and RPE/choroid of wild-type (+/+), +/N and homozygous (N/N) Best1^Y227N^ mice. *Best1* expression in normal testis from B6/J and CD-1 served as a reference. Expression in RPE/choroid and retina tissue from +/+, +/N and N/N mice of B6/J or CD-1 background repeatedly was comparable between genotypes but was rather weak by visual inspection (Fig. 2A). Regardless of the strain, western blot analysis with a well-established polyclonal C-terminal antibody, termed α-Best1-C45 (Milenkovic et al., 2015), failed to detect Best1 protein in the ocular tissues of +/+, +/N and N/N mice (B6/J and CD-1, respectively) (Fig. 2B). Instead, only non-specific staining was observed in the murine RPE. Our results suggest that normal and mutant Best1 protein is below detection level in the mouse eye.

### Retinal histology and metabolism of Best1^Y227N^ mice

**Fig. 3.**
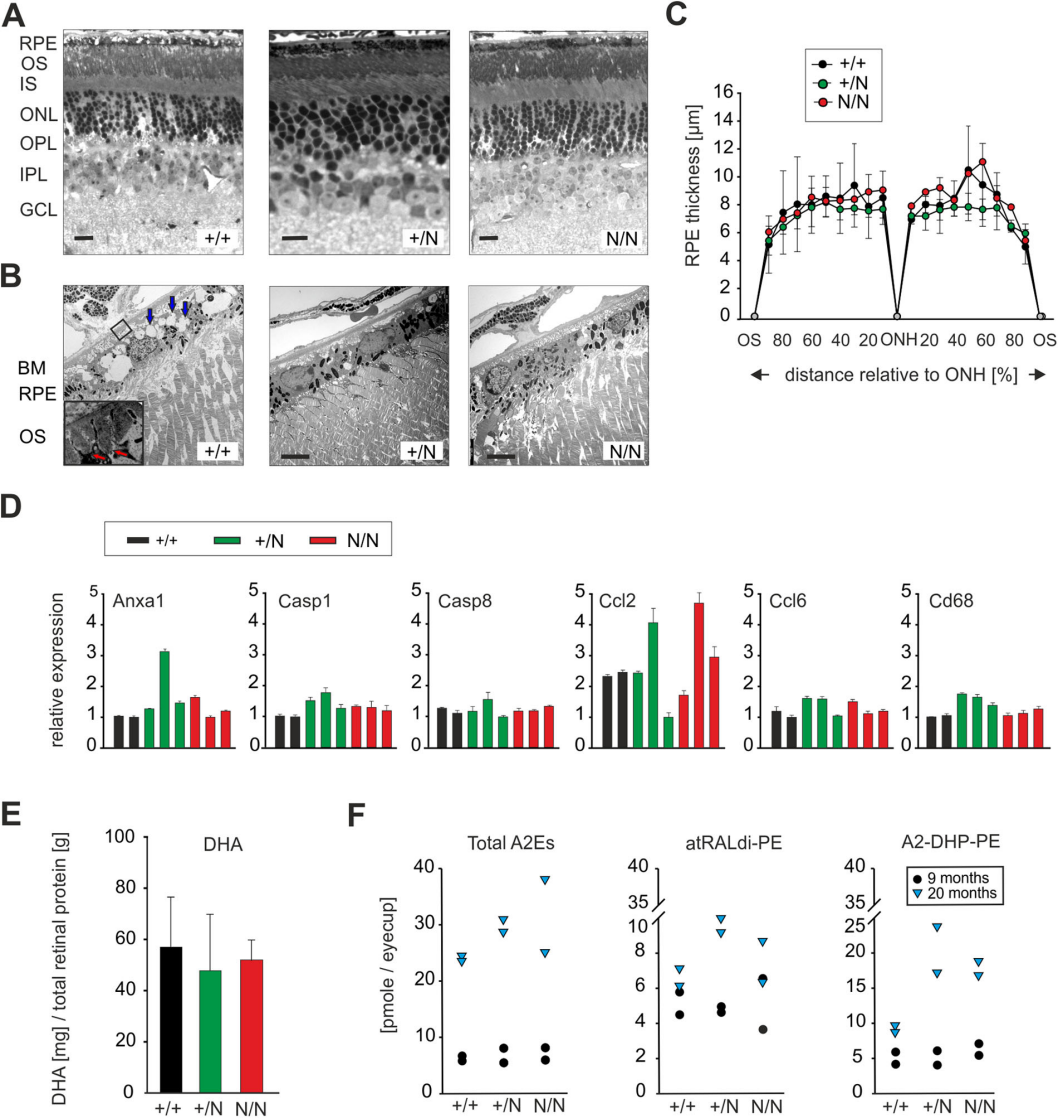
**Analysis of retinal histology and metabolism in Best1^Y227N^ mice.** (A) Light microscopy of retinal semi-thin sections of B6/J +/+, +/N and N/N mice aged 18–21 months. OS, outer segments; IS, inner segments; ONL, outer nuclear layer; OPL, outer plexiform layer; IPL, inner plexiform layer; GCL, ganglion cell layer. Scale bars: 20 μm. (B) Ultra-thin sections showing the RPE layer with associated POS; red arrows (inset), laminar infoldings; blue arrows, cytoplasmic vesicles. Scale bars: 5 μm. BM, Bruch′s membrane. (C) Quantification of RPE thickness in +/+, +/N and N/N mice. Retinae were divided in ten sections anterior and posterior of the optic nerve head (ONH), ending at the ora serrata (OS). The mean±s.d. is given for each sample; *n*=2–5 mice per genotype, two retinal sections were analyzed per mouse. (D) Quantitative real-time expression of genes involved in apoptosis and/or the activation of microglia and macrophages. *Anxa1*, Annexin 1; *Casp1*, Caspase 1; *Casp8*, Caspase 8; *Ccl2*, C-C motif chemokine ligand 2; *Ccl6*, chemokine C-C motif ligand 6; *Cd68*, CD68 antigen. Expression was normalized to Hprt1 (hypoxanthine phosphoribosyltransferase 1). RNA was extracted from the RPE/retina complex isolated from eyes of two B6/J +/+ and three +/N and N/N mice, respectively (each animal 12–13 months of age). The mean±s.d. is given for each sample. All samples were performed in triplicates. Also, see results for genes *Gfap*, *Mrs2*, *Stab1* and *Tmem2* in Fig. S1. (E) Measurements of DHA levels were performed by GC-mass spectrometry (MS) of retinal lysates from 10–12 month-old CD-1 +/+, +/N and N/N mice. *N*=8–12 mice per genotype (F) Levels of total A2E, atRALdi-PE and A2-DHP-PE as a function of age were examined by HPLC-analysis in 9- and 20-month-old B6/J +/+, +/N and N/N mice. Data points represent values from six pooled eye cups per time point and genotype.

Histological analysis of retinal cross-sections was done from 18–21-month-old B6/J wild-type and mutant mice, respectively (Fig. 3A and B). Using light microscopy, we found no noticeable differences in the retinal layers between B6/J +/+, +/N and N/N mice (Fig. 3A), although retinal detachment was observed in some regions of the eye in all *Best1* genotypes. Electron microscopy of ultra-thin sections confirmed the integrity of the RPE layer, Bruch's membrane and the underlying choroid (Fig. 3B and C). More specifically, calculations of the mean RPE thickness in the central and peripheral retina revealed no differences between the genotypes (Fig. 3C). In some mice (wild type and mutant), we occasionally found evidence of basal laminar infoldings (Fig. 3B) and a number of cytoplasmic vesicles (Fig. 3B) within the RPE layer possibly representing signs of cell aging. To further analyze the consequences of mutant Best1 on the aging RPE/photoreceptor complex, we analyzed mRNA expression of ten apoptosis-related genes (Table S1) in 13-month-old B6/J wild-type and mutant animals. These genes are known to be involved in the activation of inflammation, apoptosis and cellular senescence. Quantitative real-time RT-PCR (qRT-PCR) revealed no statistical differences between mRNA expression levels in eyes from B6/J +/+, +/N and N/N mice (Fig. 3D; Fig. S1).

We next investigated two key metabolites of retinal/RPE tissue, docosahexaenoic acid (DHA) and lipofuscin. Permanent recycling of DHA between the RPE and the inner segments of the photoreceptors maintains the functionality of the RPE/retina interface while DHA insufficiency is associated with alterations in retinal function (SanGiovanni and Chew, 2005). DHA levels in retinal lysates from CD-1 +/+, +/N and N/N mice by GC-MS fatty acid profiling were similar between the genotypes (Fig. 3E). Lipofuscin is a major RPE pigment that forms in photoreceptor outer segments and accumulates in the RPE with age. It is known to cause RPE and photoreceptor degeneration at high levels (Sparrow et al., 2012). Using HPLC analysis, we analyzed three bis-retinoid constituents of RPE lipofuscin, namely A2E (N-retinylidene-N-retinylethanolamine), atRALdi-PE (all-trans-retinal dimer-phosphatidyl-ethanolamine) and A2-DHP-PE (A2-dihydropyridinephosphatidylethanolamine), in eye cup extracts of B6/J mice aged 9 and 20 months, respectively. As expected and in agreement with previous reports (Ueda et al., 2016), we found considerably higher levels of each of the three lipofuscin compounds in aged mice (20 months) compared to the younger group (9 months) (Fig. 3F). Within the younger age-group, the concentrations of all three lipofuscin pigments did not differ significantly between the genotypes. However, all of the three lipofuscin compounds analyzed showed a trend towards higher concentration in aged B6/J +/N and N/N mice compared to age-matched B6/J +/+ controls, although concentrations varied considerably among the elder group while data failed to reach statistical significance.

### Visual function in Best1^Y227N^ mice

**Fig. 4.**
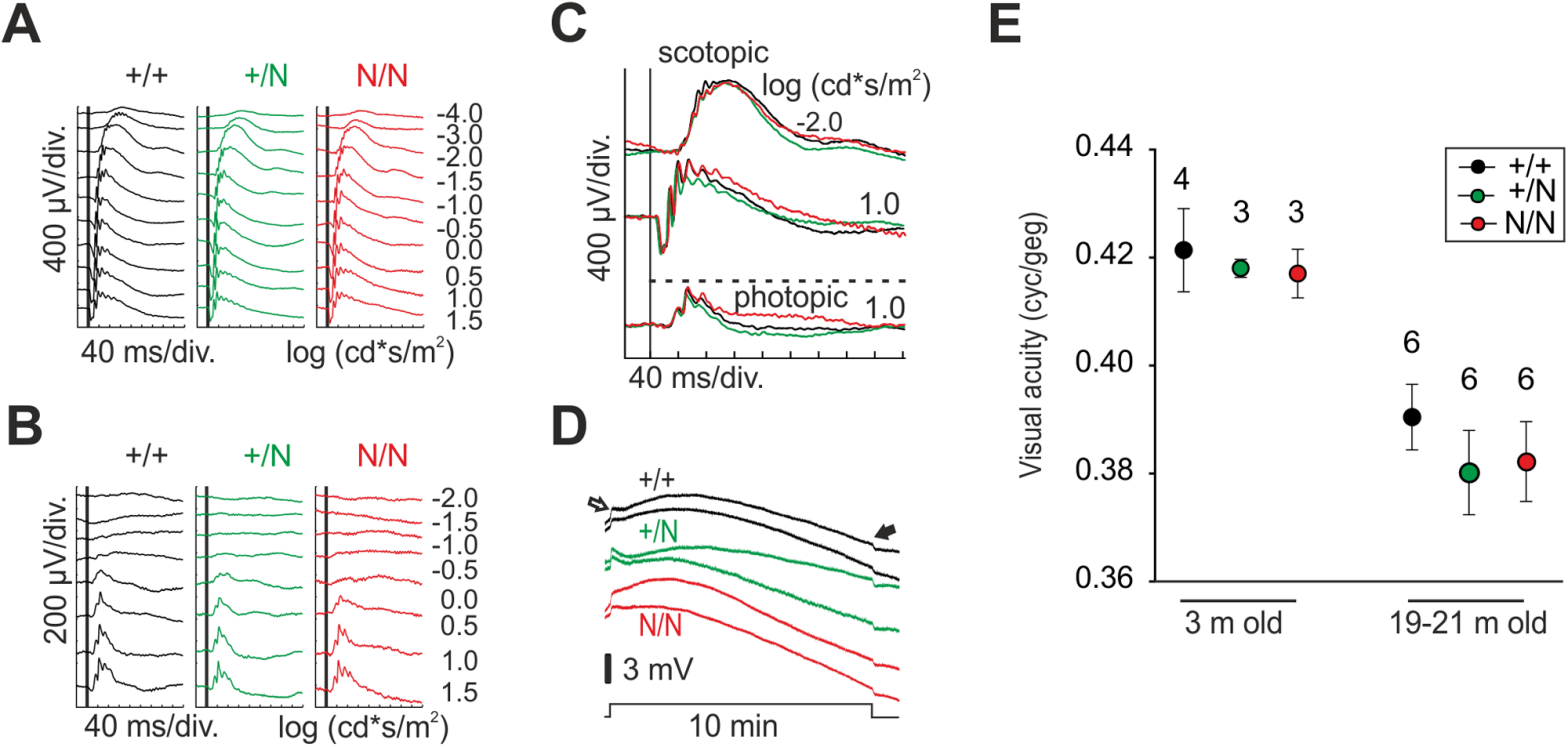
**Visual and RPE phagocytic function in Best1^Y227N^ mice.** (A–D) Scotopic and photopic electroretinogram (ERG) analysis of dark-adapted (A) and light-adapted (B) B6/J +/+, +/N and N/N mice with stimulus intensities ranging from −4–1.5 and −2–1.5 log cd*s/m^2^, respectively. (C) Overlay of indicated traces under scotopic and photopic conditions extracted from A and B. (D) Measurements of standing potential changes were performed in B6/J +/+, +/N and N/N mice using a 10 min light pulse with a fixed intensity of 0.6 log cd*s/m^2^ in fully dark-adapted animals. Shown are traces of 11 min duration using direct-current signal amplification. Arrows indicate beginning and end of light exposure. (E) Visual acuity of B6/J +/+, +/N and N/N mice was analyzed by a virtual optomotor system. As indicated, mice of two different age groups were tested five times on subsequent days for acuity of the right and left eye. Data are given as mean acuity of two eyes per mouse. *N*=number of animals analyzed.

BD is characterized by a diminished EOG light peak with a normal electroretinogram (ERG) eventually leading to visual impairment (Blodi and Stone, 1990; Marmorstein et al., 2009). To assess visual function, we first analyzed ERG measurements in Best1^Y227N^ mice. Single flash ERG responses of 6-month-old B6/J mice recorded under dark-adapted (Fig. 4A) and light-adapted (Fig. 4B) conditions using varying stimulus luminance intensities revealed no differences in retinal function among the three *Best1* genotypes. Similarities in amplitude and waveform are highlighted in selected overlays of the three genotypes under scotopic and photopic conditions (Fig. 4C). To monitor the EOG light peak, we recorded the transepithelial standing potential across the RPE of B6/J +/+, +/N and N/N mice during light adaption using direct-current signal amplification of the ERG. For this purpose, the potential of fully dark-adapted 9-month-old B6/J animals was directly traced in response to a 10 min light stimulus at a fixed intensity (0.6 log cd/m^2^), a stimulus luminance intensity similar to that used in human EOG recordings. Consistent with the characteristics of a light peak, the amplitude of the potential slowly increased during the period of light adaption although the final plateau was mostly lower than the pre-stimulus baseline (Fig. 4D). Amplitudes, waveform and stimulus for on and off responses in B6/J +/N and N/N revealed no significant difference from those of B6/J +/+ mice.

To further assess the impact of the Y227N mutation on visual function, we measured visual acuity of 3-month and 19–21-month-old B6/J mice using a virtual optomotor system as published earlier (Prusky et al., 2004). Mice were set on a platform surrounded by a rotating cylinder. By tracking the drifting gratings, reflexive head movements of the mouse were monitored and analyzed once a day for five consecutive days. Although we found an age-related decline in visual acuity when comparing the two age groups, visual acuity within the two groups was similar regardless of genotype (Fig. 4E). These findings suggest that the Best1-Y227N mutation has no major effect on visual competence in the mouse ocular tissues.

### RPE phagocytosis in Best1^Y227N^ mice

**Fig. 5.**
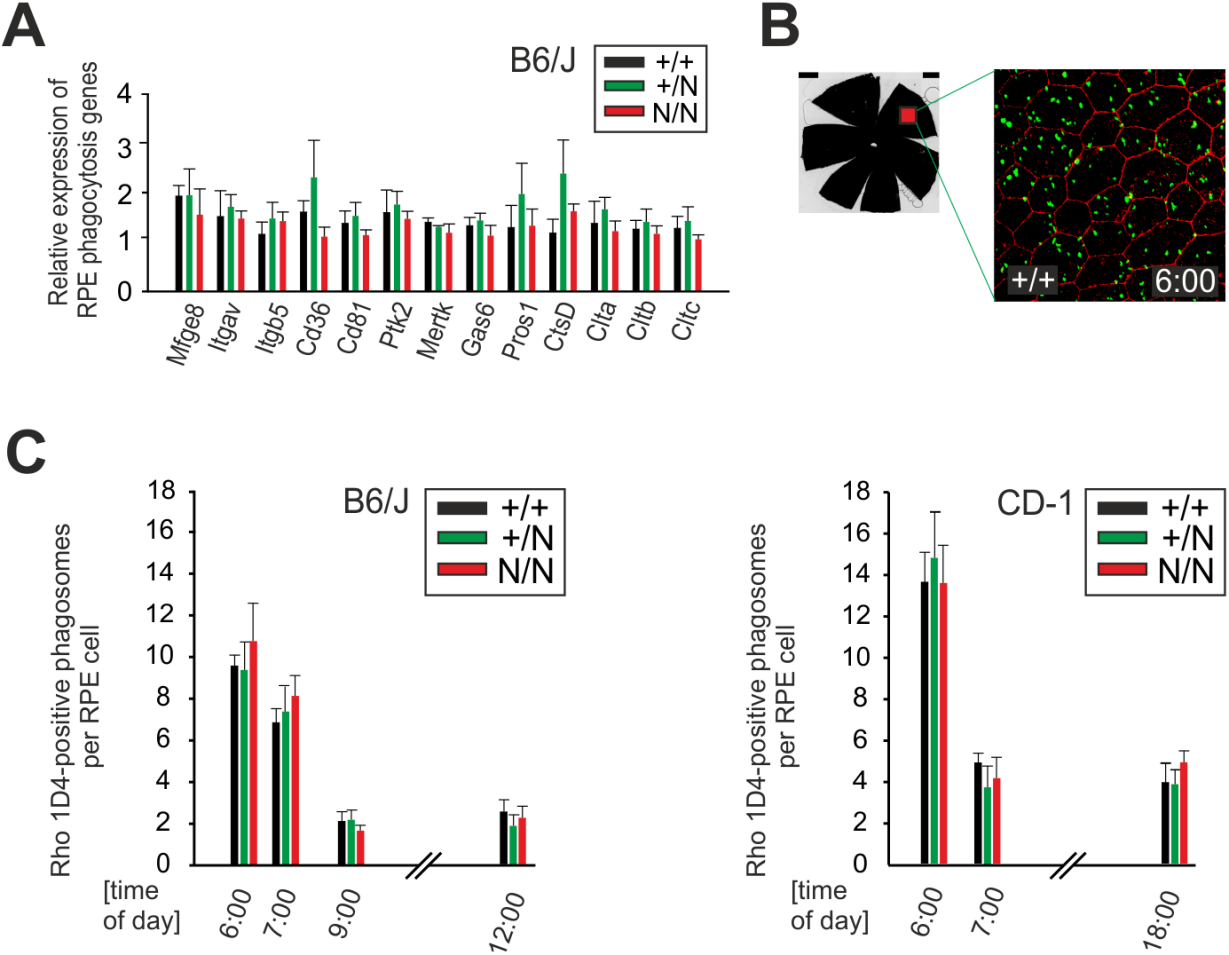
**RPE phagocytic function in Best^Y227N^ mice.** (A) Quantitative real-time expression analysis of RPE genes involved in different steps of RPE phagocytosis at 6:00am associated with POS binding (*Mfge8*, Milk fat globule-EGF factor 8 protein; *Itgav*, αV Integrin; *Itgb5*, β5 Integrin; *Cd36*, *CD36* molecule; *Cd81*, CD81 antigen) and those associated with internalization and degradation of POS (*Gas6*, Growth arrest specific 6; *PTK2*, Protein Tyrosine Kinase 2; *MerTK*, Mer Tyrosine Kinase; *Pros1*, Protein S1; *Ctsd*, CathepsinD and *Clta*, -b and -c, Clatherin light chains a, b and heavy chain). Expression was normalized to *Hprt1*. RNA was extracted from pooled RPE cells, enzymatically isolated from eyes of six B6/J +/+, +/N and N/N mice, respectively. The mean±s.d. is given for each sample. All samples were performed in triplicates; *n*=12 eyes from six mice per genotype. (B) Representative flat mount (left image) from a 3-month-old B6/J +/+ mouse showing a photographic field (red rectangle). Rho-1D4 staining (green) of POS in a representative photographic field of RPE flat mounts, shortly after light onset at 6:00am. Cell boundaries were determined by red staining with ZO-1 (right image). (C) Quantitative analysis of diurnal RPE phagocytosis of shed photoreceptor outer segments (POS) of +/+, +/N and N/N mice with the B6/J (left graph) and CD-1 (right graph) genetic background, respectively. The mean of 1D4-positive phagosomes per RPE cell is given from six to eight photographic fields of immunostained flat mounts at the indicated time points. The mean±s.d. is given for each time point; *n*=4 eyes from two mice. Also, see results from Rho-4D2 immunostaining in Fig. S2.

Disk shedding is a most important function of the RPE to ensure continuous renewal of the light-sensitive rod and cone outer segments of photoreceptors (Young, 1967). This process follows a strict diurnal rhythm (LaVail, 1980). To examine the consequences of mutant Best1 on POS shedding and degradation of shed tips by phagocytosis, we first performed qRT-PCR of 13 phagocytosis-related genes. These genes are known to be involved in RPE phagocytosis during the so-called diurnal burst, shortly after light onset at 6:00am and are associated with the binding, internalization and degradation of POS in the RPE/choroid complex (Mazzoni et al., 2014). There was no statistical difference between the mRNA expression levels in B6/J +/+, +/N and N/N mice (Fig. 5A).

To further analyze engulfment and clearance of POS by the RPE in the diurnal course of retinal phagocytosis, we immunostained RPE flat mounts with antibody Rho-1D4 (1D4) specifically recognizing the C-terminus of rhodopsin. This facilitates the quantification of phagosomes containing 1D4-positive POS components at different time points relative to light onset. Consequently, fluorescence microscopy revealed a high number of 1D4-positive phagosomes at the apical RPE cell layer of B6/J and CD-1 +/+, penetration depth indicated by staining of the tight junction protein ZO-1, confirming regular POS internalization at 6:00am (Fig. 5B). The phagocytic burst is followed by a rapid decrease in the number of 1D4-positive phagosomes in a time-course of 1–12 h after light onset, representing efficient phagolysosomal digestion that is in line with previous reports (Damek-Poprawa et al., 2009). Quantification of phagosome numbers at defined time points in the RPE of B6/J and CD-1 +/+, +/N and N/N mice revealed comparable numbers of 1D4-positive phagosomes at all time points of the day (Fig. 5C). Of note, there was also no difference in the amount of immunostained POS between genotypes when using an antibody against the N-terminal epitope of rhodopsin (Rho-4D2) (Fig. S2). Taken together, these results indicate that the introduction of the Y227N mutation does not interfere with POS uptake, RPE phagocytosis and processes associated with circadian rhythm.

### Expression profiling of mutant Best1 in the mouse testis

**Fig. 6.**
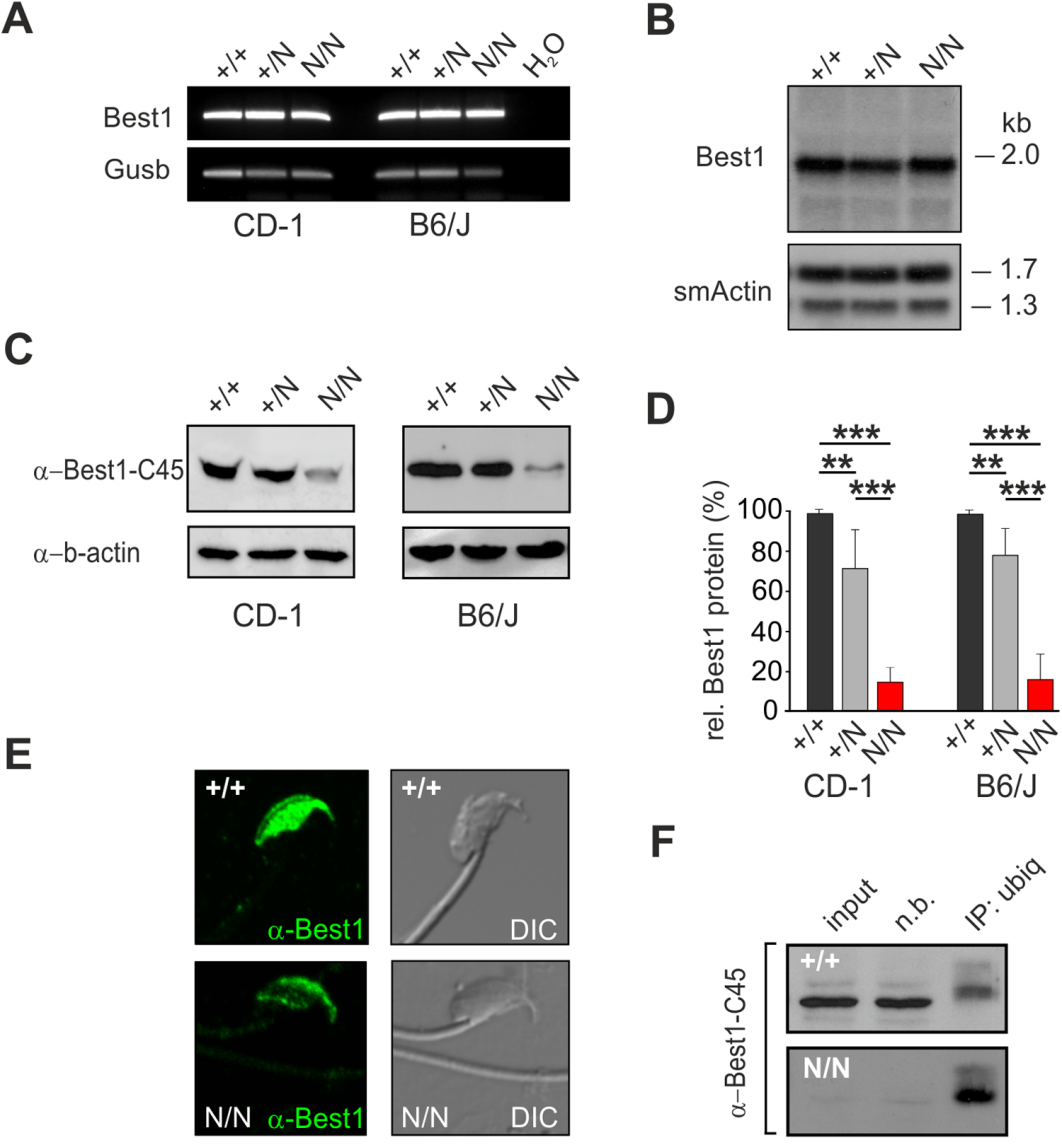
**Analysis of mutant Best1 protein (Y227N) in mouse testis.** (A) RNA expression of *Best1* in testis from CD-1 (left graph) and B6/J (right graph) +/+, +/N and N/N mice by RT-PCR. *Gusb* served as control for RNA integrity. (B) Northern blot analysis of total RNA from B6/J +/+, +/N and N/N mouse testis by hybridization to a probe targeting *Best1* exon 7–10. Mouse smooth muscle actin served as control for equal loading and RNA integrity. (C) Representative western blot of whole testis lysates from CD-1 (left graph) and B6/J (right graph) +/+, +/N and N/N mice using α-Best1-C45. Anti-beta actin served as loading control. (D) Quantification of Best1 protein expression relative to CD-1 and B6/J +/+, respectively (*n*=4 mice of each genotype) obtained from C (data were extracted from two to three western blots of each triplet; one-way ANOVA; **P<0.01; ***P<0.001). (E) Immunofluorescence staining of sperm cells from B6/J +/+ and N/N males using α-Best1-C45. (F) Testis tissue from B6/J +/+ and N/N mice was immuno-precipitated with α-ubiquitin, transferred to nylon membranes and probed as indicated using α-Best1-C45 (n.b., fraction not bound). For each sample, the mean±s.d. is given.

RT-PCR, northern blot and western blot analysis was done in testis tissue from B6/J and CD-1 +/+, +/N and N/N mice. RT-PCR revealed no differences in mRNA expression in testis derived from the various genotypes (Fig. 6A). For confirmation, northern blot analysis was performed and showed a single transcript of ∼2 kb in testis mRNA of all three genotypes confirming strong and comparable *Best1* expression of full-length *Best1* mRNA in B6/J +/+, +/N and N/N mice (Fig. 6B). In line with previous data (Milenkovic et al., 2015), western blot analysis confirmed prominent Best1 protein expression in lysates from B6/J +/+ and CD-1 +/+ testis (Figs 6C and 2B). In contrast, Best1 protein expression was severely reduced in B6/J and CD-1 N/N (<18% of remaining Best1 protein) and to a lesser extent in B6/J +/N and CD-1 +/N mice (<80%) compared to B6/J +/+ and CD-1 +/+ controls (Fig. 6C and D), suggesting severe degradation of mutant Best1 protein. This was consistent with a reduced immunostaining of Best1 protein in the B6/J N/N sperm head in contrast to B6/J +/+ animals where immunolabeling consistently stained the equatorial segment of the sperm head as reported earlier (Milenkovic et al., 2015) (Fig. 6E).

To examine whether reduced Best1 protein in mouse testis is caused by enhanced protein degradation, we examined ubiquitination in testis lysates from B6/J +/+ and B6/J N/N males as conjugation of a single ubiquitin to a protein can trigger degradation of the tagged protein (Terrell et al., 1998). Cell lysates were immunoprecipitated with α-ubiquitin and immunoblotted with α-Best1-C45 antibody solution. Western blot analysis identified a faint single immunoreactive Best1 molecular weight staining in +/+ testis, approximately ∼8 kDa above the unmodified protein (Fig. 6F), consistent with Best1 being mono-ubiquitinated, and tagged for endo-lysosomal degradation (Haglund et al., 2003). In contrast to B6/J +/+, testis lysates from B6/J N/N males showed increased amounts of mono-ubiquitinated Best1. Together, these findings indicate that reduced amounts of endogenous Best1 protein in the B6/J N/N mouse are likely resulting from decreased protein stability rather than reduced transcription.

### Sperm motility and fertilization rates in Best1^Y227N^ mice

**Fig. 7.**
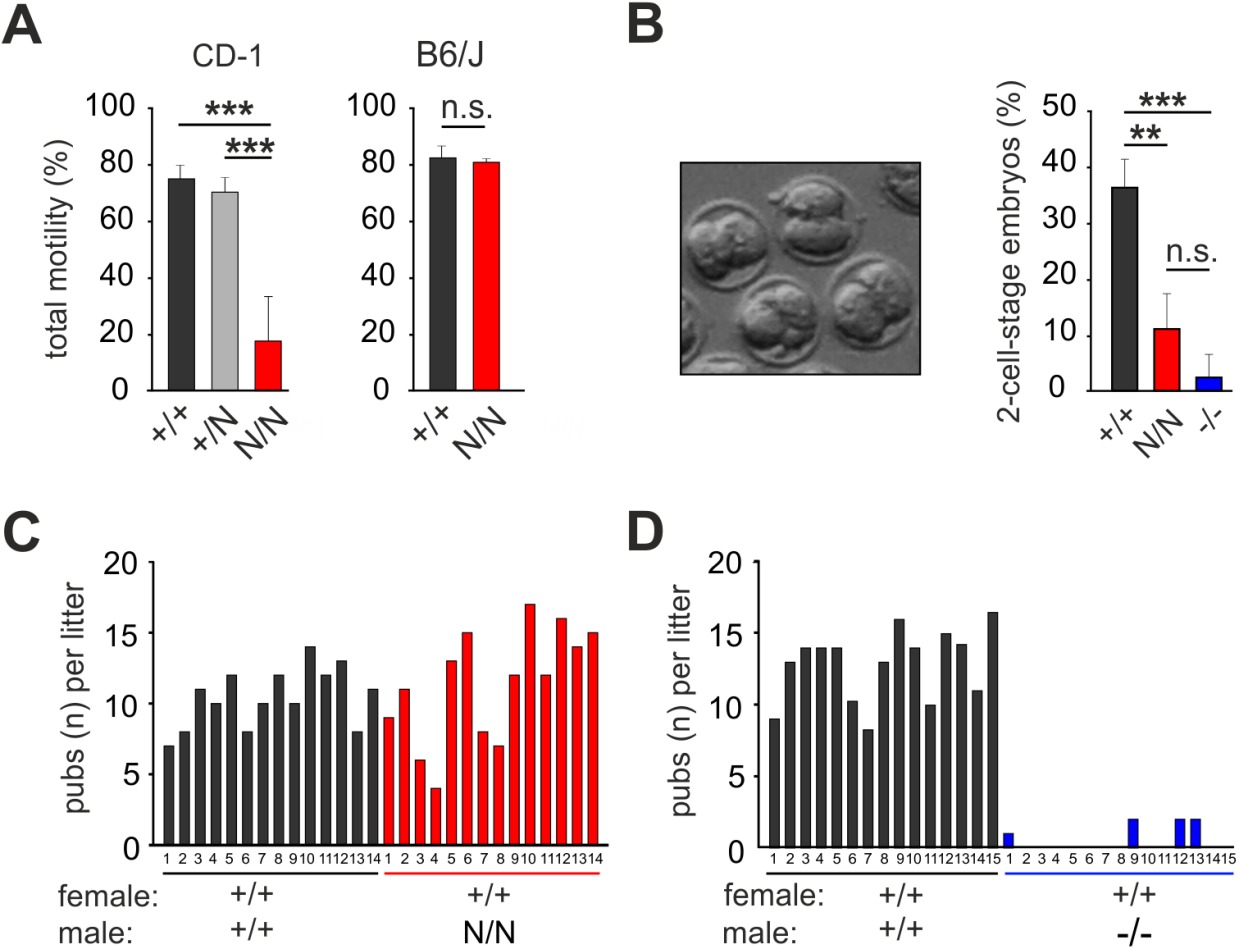
**Analysis of mutant Best1 protein (Y227N) in testis and sperm function.** (A) Percentages of total sperm motility from caudal sperm of CD-1 +/+, +/N and N/N (left graph) (*n*=6 mice for each genotype; one-way ANOVA, **P<0.01; ***P<0.001) and B6/J (right graph) +/+ and N/N mice (*n*=4 and six mice, respectively; two-sided paired Student's *t*-test; n.s., not significant). (B) Phase contrast image of two-stage eggs from a CD-1 +/+ mouse after IVF and quantitative analysis of eggs fertilized with sperm from CD-1 +/+, N/N and −/− males. (C,D) Reproductive capacity of CD-1 females mated with sperm from CD-1 N/N (C) and CD-1 −/− (D) mice and the corresponding +/+ littermates. For each sample, the mean±s.d. is given.

Recently, we showed that complete loss of Best1 protein in the Best1 knockout mouse impairs sperm function and results in a severe sub-fertility phenotype (Milenkovic et al., 2015). To examine the functional consequences of reduced Best1 protein expression in the +/N and N/N testis, we analyzed total motility from caudal sperm after release into Toyoda Yokoyama Hoshi (TYH) media as well as their capability to fertilize oocytes *in vitro* (IVF). While total motility of sperm from B6/J N/N mice was unaffected (Fig. 7A), we found a significant lower percentage of motile sperm in CD-1 N/N mice (20±15%) compared to CD-1 +/+ (75±6%) and CD-1 +/N males (70±6%) (Fig. 7A). This resulted in significantly lower fertilization rates when oocytes were incubated with sperm from CD-1 N/N (11% fertilized eggs) compared to CD-1 +/+ (36%) (Fig. 7B). In comparison, sperm from CD-1 Best1-deficient mice (−/−) revealed even lower fertilization rates with only 2% of fertilized eggs (Fig. 7B). This may explain why normal CD-1 females mated with CD-1 N/N males give birth to a normal number of pups (mean litter size 13±3.4 pups) (Fig. 7C) in contrast to normal females mated with CD-1 −/− males (<0.5%) as reported earlier (Milenkovic et al., 2015) (Fig. 7D).

## DISCUSSION

Mouse models of inherited retinal dystrophy have regularly been shown to be valuable tools for the analysis of molecular disease mechanisms but also for evaluating efficacy of novel therapeutic approaches (Chan et al., 2004; Isgrig et al., 2017). Here, we report the generation and characterization of a gene-modified mouse line carrying the human BD-associated mutation Y227N in the endogenous murine *Best1* gene. Although this gene mutation in the human homologue is well established to cause autosomal dominant BD, we show that the mouse fails to reveal any of the typical features of human BEST1-associated retinopathy but instead exhibits strong effects on protein stability in the testis and consequently on sperm motility. To this end, the sperm-related phenotype offers an excellent *in vivo* model to evaluate pharmacological modulation of Best1 protein expression and function as a potential strategy for treatment of BEST1-associated disease.

The absence of functional defects in ocular tissue of Best1-modified mice, even in mutant mice up to 21 months of age, may be viewed in a more general debate of whether the nocturnal mouse is in fact a relevant model for macular degeneration in diurnal human. Of course, the mouse is known to be coneless with no anatomically comparable macular area. Nevertheless, Volland et al. demonstrated a greater density of photoreceptors in the mouse central retina than in the human macula suggesting that the phagocytic load is even higher in the mouse RPE (Volland et al., 2015). In line with these observations, a number of mouse models show remarkable overlaps with human retinal dystrophies, despite the fact that initial pathological damage is known to arise preferentially in the human macula (e.g. Weber et al., 2002a,b; Marmorstein et al., 2007). The absence of an ocular/retinal phenotype in the Best1^Y227N^ mouse, even in two genetically divergent backgrounds, may simply be explained by findings in this study and by others that normal as well as mutant Best1 protein is below immunohistochemical detection in the murine RPE which is in striking contrast to the human situation (Mullins et al., 2005). This peculiarity in species-specific expression suggests that Best1 likely plays no crucial role in the murine eye. Nevertheless, Zhang et al. reported a knock-in mouse with a mutation at W93C, known to cause BD in human. Similar to our Y227N mutant mouse, the W93C mutation was also crossed onto the B6/J genetic background (Zhang et al., 2010). In contrast to the present study, these mice were reported to show some features of BD, including a deficit in the light peak, serous retinal detachment, accumulation of lipofuscin and abnormalities in calcium signaling in RPE cells. Interestingly, homozygous and heterozygous Best1-W93C mice (Zhang et al., 2010) but also Best1-deficient animals (Marmorstein et al., 2006) failed to reveal aberrant chloride currents as would be expected from earlier studies demonstrating that the introduction of mutation W93C diminishes human BEST1 (hBEST1) associated anion conductance (Sun et al., 2002; Lee et al., 2010; Marmorstein et al., 2015). Taken together, these inconsistencies suggest that the phenotypic abnormalities seen in the Best1^W93C^ mouse may not be the result of impaired Best1 channel activity, although reasons to explain the different phenotypic retinal presentation of mutant mice harboring the Y227N or the W93C mutation are still unclear. It is possible though that the two mutations may have different effects on localization or stability of the mutant protein. While both, Y227N and W93C are known to mis-localize in Madin-Darby canine kidney (MDCKII) cells (Milenkovic et al., 2011, 2018), recent work from Johnson et al. (2013) demonstrated that the presence of properly localized wild-type Best1 can rescue the mis-localization caused by W93C. The Y227N mutation clearly reveals a dominant-negative effect (Mullins et al., 2005) as do most BEST mutations analyzed so far (Johnson et al., 2014). Unfortunately, the spurious Best1 expression in the murine RPE prevents a more thorough immunohistochemical examination of endogenous normal Best1 but also of mutant proteins Y227N and W93C.

In this study, we confirmed previous results demonstrating that Best1 protein is readily detectable in mouse testis (Milenkovic et al., 2015). Analysis of testis protein extracts from homozygous B6/J and homozygous CD-1 Best1-Y227N mice demonstrated a severe decrease in Best1 protein consistent with a number of *in vitro* studies (Uggenti et al., 2016; Marmorstein et al., 2018; Milenkovic et al., 2018). These findings suggest that a reduced protein stability and enhanced protein degradation of mutant protein play a key role in BD pathogenesis. In support of this, our *in vivo* data also demonstrate a reduced Best1 protein expression strongly arguing that this is due to increased protein degradation rather than to defective protein synthesis as *Best1* mRNA expression is comparable in testis of +/+, +/N and N/N mice. Also a covalent attachment of a single ubiquitin molecule, as found in this study, potentially targets mutant Best1 protein for lysosomal degradation (Haglund et al., 2003). Our recent *in vitro* study is in agreement with this conclusion where mis-localized and unstable BD-associated mutants are degraded via the endo-lysosomal degradation pathway (Milenkovic et al., 2018). Still, ultimate proof for an exclusion of a defective Best1 protein synthesis would require the inhibition of the underlying protein degradation pathway in cell lines of testicular somatic cells from Best^Y227N^ mice. Sperm cells, however, are not suited for appropriate cell culture experiments.

Phenotypic differences in mouse models due to their respective genetic background are well established (Eshraghi et al., 2016). To this end, we crossed the Best1Y227N mutation to an inbred as well as a genetically more diverse outbred mouse strain. For the latter, we decided on the CD-1 strain as these mice are known to be robust and generally are characterized by a large litter size. In addition, the albino phenotype of the CD-1 strain is thought to serve as a susceptible background to eventually enhance a (weak) retinal phenotype introduced by genetic manipulation (Montalbán-Soler et al., 2012). Nevertheless, the inclusion of CD-1 backcrossed knock-in mice in some critical aspects of retinal metabolism and function (e.g. DHA measurements and RPE phagocytosis) did not reveal any detectable retinal abnormalities despite the fact that the outbred CD-1 background with the albino eye phenotype should particularly be susceptible to genetic damage (Montalbán-Soler et al., 2012). In contrast, we show that the sperm phenotype in homozygous knock-in mice is more severe in the CD-1 than the B6/J background. While CD-1 and B6/J knock-in mice show a comparable low level testicular Best1 protein expression, the effects on sperm motility differ tremendously between the two strains. This may be best explained by background-specific modifiers and is in line with our results from Best1-deficient mice, where homozygous B6/J Best1-knockout males produced ∼40% of pups of wild-type littermates, while on a CD-1 background Best1-deficient males were almost infertile (<0.5%) (Milenkovic et al., 2015).

In summary, here we report on a Best1-modified knock-in mouse line harboring the human Y227N mutation in the homologous murine gene. Mutant mice reveal no ocular/retinal phenotype but manifest a strong testicular effect on protein stability and sperm motility that is greatly modified by genetic background. Our data suggest that the observed phenotype in testis is due to increased protein degradation rather than defective protein synthesis. Due to this strong phenotype, our mouse strain offers an excellent *in vivo* model to analyze therapeutic approaches aiming at testing drug efficacies of compounds modifying protein stability and the degradative processes of mutant BEST1 protein. In a note of caution, it should be mentioned that pharmacological intervention in a tissue other than the one harboring the primary pathology (e.g. the RPE in BD) may be error prone as testicular protein degradation mechanisms may differ from those of the RPE. Further experiments will be needed to validate the utility of such an approach.

## MATERIALS AND METHODS

### Antibodies

Rabbit polyclonal antibodies included Best1-C45 (Milenkovic et al., 2015), diluted 1:5.000 for WB and 1:500 for ICC; ZO-1 (#61-7300, Thermo Fisher Scientific, Waltham, USA), diluted 1:250; mouse monoclonal antibodies against β-actin (#5441, Sigma-Aldrich, Munich, Germany), diluted 1:1.000; Rpe65 (ab13826, Abcam, Cambridge, UK), diluted 1:5.000; Rs1, Rho-4D2 and Rho-1D4 (kindly provided by Dr Robert Molday, University of British Columbia, Vancouver, Canada), diluted 1:5.000, 1:2.500 and 1:10.000, respectively. Secondary antibody for immunofluorescence detection was goat Alexa 488 or 594-conjugated anti-rabbit and goat Alexa 594-conjugated anti-mouse (Thermo Fisher Scientific, dilution 1:500). Western blot experiments were performed with horseradish peroxidase-conjugated secondary antibodies (Calbiochem/Merck, Darmstadt, Germany; dilution 1:10,000).

### Immunofluorescence labeling

Immunofluorescence was performed as previously described (Milenkovic et al., 2015). In brief, a 20 μl drop of sperm suspension was air-dried on microscope slides and stained with α-Best1-C45 at 4°C overnight (ON). Immunostained spermatozoa were imaged on a Zeiss confocal microscope LSM 510 (Zeiss, Göttingen, Germany).

### Sample collection and preparation for RNA and protein expression

Testis tissue was cut into small pieces and homogenized using a TissueLyzer (Qiagen, Hilden, Germany). For preparation of RPE/choroid and retina samples, eyes were enucleated, bulbi were incised along the ora serrata and cornea, lens, iris and vitreous body were removed. After incubating the posterior eye cup in phosphate buffered saline (PBS, pH 7.4) at 37°C for 20 min the retina was removed. For western blot analysis and RT-PCR eye cups (sclera, RPE and choroid) and retinae were then homogenized using a TissueLyzer. For qRT-PCR, eye cups were treated enzymatically to isolate pure RPE cells. After removal of the retina, eye cups were incubated 30 min in PBS/1 mM EDTA, then transferred to a dissociation buffer of 3 mM L-cysteine (Sigma-Aldrich) in PBS/1 mM EDTA, 1 U/ml papain (Sigma-Aldrich), and 1 mg/ml BSA (Sigma-Aldrich) for 23 min. RPE cells were rinsed in DMEM culture medium (Thermo Fisher Scientific) supplemented with 2% FCS to stop the reaction (Thermo Fisher Scientific). After a washing step with PBS the isolated RPE cells were pelleted and subsequently stored at −80°C.

### Protein sample preparation, SDS page and quantitative western blot analysis

Whole cell protein samples were prepared by homogenization in a modified RIPA buffer (20 mM Tris/HCl, 150 mM NaCl, 1 mM EDTA, 1 mM EGTA, 0.2% DOC, 0.25% NP 40, 0.025% SDS) supplemented with 1× Protease inhibitor cocktail (Roche, Mannheim, Germany). Protein extracts were sheared by passing through a 25-G needle several times on ice and subsequently with seven 1-s pulses at 32% amplitude using a Vibra-Cell sonicator (Sonics, Newtown, USA). Protein concentration was determined on a NanoDrop™ (Thermo Fisher Scientific) and equal amounts of protein were separated on SDS-Page and subsequently transferred onto Immobilon^®^-P PVDF membrane (Millipore, Bedford, USA). Incubation of primary and secondary antibodies was carried out at 4°C overnight. Protein labeling was visualized by chemiluminescence using the Odyssey^®^ Fc Imaging System.

### RNA isolation, reverse transcription and RT-PCR

Total RNA was extracted after DNase treatment (Roche) according to the manufacturer's instructions using the RNeasy Mini Kit (Qiagen). First strand cDNA synthesis from 1 μg of total RNA was performed with RevertAid™ H Minus First Strand cDNA Synthesis Kit (Fermentas, Thermo Fisher Scientific) and random hexamer oligonucleotide primers. For RT-PCR reactions 50 ng of cDNA was used as templates for PCR with Go Taq Polymerase (Promega, Mannheim, Germany). RT-PCR amplification of mBest1 and mGus was performed using primer pairs mVMD2-cDNA-F (5′-CTG CAG GTG TCC CTG TTG T-3′)/mVMD2-cDNA-R (5′-TGT CTG AAC TGG AGG GTG CT-3′) and mGusb-ex11-F (5′-GAC CCG CCT CGC ATG TTC AG-3′)/mGusb-ex12-R (5′-GCC CTG AAC CGT GAC CTC C-3′), respectively.

### Quantitative real-time RT-PCR

Quantitative real-time RT-PCR was performed with an ABI7900HT machine (Applied Biosystems, Darmstadt, Germany) using the 1× TaqMan Universal PCR Master Mix and dual-labeled probes (Roche ProbeLibrary, Roche Applied Science). For qRT-PCR reactions 25 ng of cDNA was used as templates for PCR. Measurements were performed in triplicates and results were analyzed with an ABI sequence detector software version 2.3 (Applied Biosystems) applying the ΔΔCt method for relative quantification. Primer sequences are listed in Table S1.

### Construction of the Best1 knock-in targeting vector

To introduce the Y227N mutation into exon six of the murine *Best1* gene, a modified 5′-murine fragment, spanning exon 3 to exon 6, was generated by site-direct mutagenesis and co-ligated with an unmodified 5′-fragment into the pGADT7 vector. The 3′-fragment, spanning *Best1* intron 6 to intron 9, was inserted into the ClaI site of the pGADT7 vector. A neomycin phosphotransferase (neo) gene flanked by loxP sites was integrated between the two arms. The final knock-in construct was linearized with restriction enzyme XmaI.

### Homologous recombination in ES cells and generation of germ line chimeras

CJ7 ES cells were electroporated and ES cell clones were selected and screened for homologous recombination as previously described (Swiatek and Gridley, 1993). Recombinant ES cells were injected into B6/J blastocysts. Chimeric founders were bred to B6/J mice, and heterozygous mice were backcrossed into the inbred B6/J or outbred CD-1 (CD-1^®^ IGS) genetic background (Schrewe et al., 1994; Weber et al., 2002a) (Charles River Laboratories, Sulzfeld, Germany). Genotyping of animals was performed by PCR amplification using primer pair mVMD2-NdeI-F (5′-GTC TAG GGA GCT GCA TAT GG-3′)/mVMD2-R7 (5′-AAC AGC CAG TTG TAC CTG AC-3′). Prior to analysis, knock-in mice were backcrossed for at least ten generations into B6/J or CD-1 strains.

For analysis of testis and sperm cells, male mice were between 2–4 months of age. Retinal tissues were used at the indicated ages. All mice were maintained on a 12 h light/12 h dark cycle, housed under specific pathogen-free conditions and generally maintained under guidelines established by the institution for their use. Mice were euthanized by cervical dislocation after inhalation of carbon dioxide.

### Histological analysis of retinal sections by light and electron microscopy

19–21-month-old +/N and N/N mice and age matched littermate control animals were fixed by intracardiac perfusion with 1% paraformaldehyde (PFA) plus 1% glutaraldehyde containing 0.2 M cacodylate buffer. Eyes were enucleated and fixed overnight in Karnovsky's buffer (2.5% glutaraldehyde, 2.0% paraformaldehyde in 0.1 M cacodylate buffer, pH 7.2), washed with 0.2 M cacodylate buffer pH 7.4 and post fixed for 2 h in 1% osmium tetroxide and embedded in EPON (Serva, Heidelberg, Germany) after dehydration. Semi-thin sections (1 μm) from the central retina were cut along the vertical meridian of the eye at the optic nerve head (ONH) and counterstained with Methylene Blue and viewed on a Zeiss Axioskop-2 microscope using the AxioVision LE Rel. 4.5. software. Quantification of whole retinal thickness was assessed by dividing retinal areas into ten sections anterior and posterior of the ONH. Ultra-thin sections (50–80 nm) were contrasted with 4% uranyl acetate in 50% EtOH and 2% lead citrate in 1 M NaOH and viewed with an electron microscope (EM 902, Zeiss).

### Analysis of retinal Docosahexaenoic Acid (DHA) concentration

DHA concentration was measured from retinae of CD-1 +/N, N/N and +/+ mice aged 10–12 months. Retinal protein concentrations were determined by a standard Bradford assay (Roti-quant^®^, Roth, Karlsruhe, Germany) after homogenization. DHA concentrations were analyzed by GC-mass spectrometry (MS). Briefly, 50 μg protein equivalents of each retinal homogenate were derivatized with acetyl chloride in methanol for 2 h at 80°C (Sparrow et al., 2012). An internal DHA standard mixture was added prior to methylation, followed by an extraction of methyl esters by hexane and analyzed using a Shimadzu QP-2010 GC-MS. DHA quantification was performed by external calibration and DHA levels are given as DHA [mg]/total retinal protein [g].

### Measurement of visual acuity

A Virtual Optomotor System (OptoMotry, CerebralMechanics, Lethbride, Canada) was used to measure visual acuity as an index of visual function (Prusky et al., 2004). For that purpose, animals were exposed to moving and wave gratings of various spatial frequencies and reflexive head movements of mice were tracked. Acuity was assessed by starting with a low spatial frequency (0.1 cycle/degree) and incrementally increasing the spatial frequency of the grating until the animal failed to respond. The threshold was defined as the highest spatial frequency obtained at 100% contrast. Each mouse was tested five times for acuity of the right and the left eye, respectively.

### Diurnal RPE phagocytosis assay on RPE flat mounts

Eyes from 8–12-month-old +/N and N/N and corresponding littermate control mice (+/+) were harvested at various time points after light onset (6:00am). After trimming the enucleated eyes, each eye was pierced once above the ora serrata (OS), prefixed by immersing in 2% PFA/PBS for 4 min at room temperature followed by incising along the OS to remove lens and vitreous body. The retina was peeled away after an incubation step in PBS for 20 min at 37°C. After further 6 min fixation in 4% PFA/PBS immunofluorescence labeling was performed using primary antibody ZO-1 and Rho-1D4 or Rho-4D2. Incisions were made from the peripheral eye cup towards the optic nerve head to gain a flat mount preparation and mounted onto glass slides. Flat mounts were imaged on a Zeiss Axioskop-2 microscope using AxioVision LE Rel. 4.1. software and confocal microscope LSM 510 (Zeiss). The number of Rho-1D4-positive phagosomes per RPE cell, defined by ZO-1 staining, was assessed using ImageJ.

### Analysis of RPE lipofuscin pigments

RPE lipofuscin pigments were analyzed from 9- and 20-month-old BL/6J +/N and N/N mice and their corresponding littermate controls as described (Kim et al., 2007; Wu et al., 2009). These measurements were performed specifically on the BL/6J background to ensure comparability to earlier studies (Zhang et al., 2010). Briefly, posterior murine eye cups including sclera, choroid, RPE and neural retina were homogenized in PBS using a glass tissue grinder and extracted in chloroform/methanol (2:1). Extracts were passed through a reverse phase cartridge with 0.1% trifluoroacetic acid (TFA) in methanol, dried under argon, re-dissolved in methanol and analyzed by reverse-phase HPLC (Waters Alliance system, Milford, MA, USA). A2E, atRAL-di-PE and A2-DHP-PE quantification was performed by external calibration. Data are given as molar quantity/eye cup.

### Northern Blot

For northern blot analysis, total RNA (10 µg) was isolated from frozen testis, electrophoretically separated on a 1,2% agarose gel containing 1% formaldehyde and blotted onto nylon membrane (Amersham, Freiburg, Germany). Hybridization probes were generated by RT-PCR encompassing exon 9–10 of the *mBest1* gene (NM_011913.2) using primer pair VMD2-Maus-cDNA-F/R (5′-TGT CTG AAC TGG AGG GTG CT-3′)/(5′-AGG GAG TAA TGG TTG GAA TGG G-3′). Mouse smooth muscle actin served as a control using primer pair mSMactin_F/R (5′-AGG GAG TAA TGG TTG GAA TGG G-3′) and (5′-CAG ACG CAT GAT GGC ATG AGG-3′). The fragments were randomly labeled in the presence of [α-32P] dCTP (Redi Prime II DNA Labeling System, GE Healthcare, München, Germany). Removal of unincorporated nucleotides was achieved by Sephadex™ chromatography.

### *In vivo* electroretinographic analysis

Electroretinograms (ERGs) were recorded binocularly according to previously described procedures (Tanimoto et al., 2012, 2015). The ERG equipment consisted of a Ganzfeld bowl, a direct current amplifier and a PC-based control and recording unit (Multiliner Vision; VIASYS Healthcare GmbH, Hoechberg, Germany). Mice were dark adapted overnight and anaesthetized with ketamine (66.7 mg/kg body weight) and xylazine (11.7 mg/kg body weight). The pupils were dilated and single flash ERG responses were obtained under dark-adapted and light-adapted conditions. Light adaptation was accomplished with a background illumination of 30 candela (cd) per square meter starting 10 min before recording. Single white-flash stimulus intensity ranged from −4–1.5 log cd*s/m^2^ under dark-adapted and from −2–1.5 log cd*s/m^2^ under light-adapted conditions, divided into ten and eight steps, respectively. Ten responses were averaged with an inter-stimulus interval of either 5 s or 17 s (for 0, 0.5, 1, and 1.5 log cd*s/m^2^). Band-pass filter frequencies were 0.3 and 300 Hz.

### *In vivo* standing potential analysis

There is a standing electrical potential across the eye, generated largely by the transepithelial potential across the RPE which depends upon the state of ambient retinal illumination (Wimmers et al., 2007). As constant potentials are difficult to measure directly due to baseline drift and movement artefacts, EOG has been developed as an indirect method in patients that are able to move their eyes between two fixation sites (Constable et al., 2018). To obtain information about the standing potential changes with light exposure in mice that are not able to move their eyes (particularly when properly anesthetized), we performed a direct recording of the potential using the same recording equipment as for the ERG (see above). Briefly, gold wire ring electrodes (active electrodes) were moistened with methylcellulose and positioned on the surface of both murine corneae for binocular ERG recordings. Stainless steel needle electrodes were applied subcutaneously at the middle of the forehead region and the back near the tail as a reference and a ground electrode, respectively. A 10 min light pulse was used at a fixed intensity (0.6 log cd/m^2^) in fully dark-adapted animals. Traces of 11 min duration (i.e. including 15 s before and 45 s after light exposure) were obtained using direct-current signal amplification (Band-pass filter frequencies were 0 and 300 Hz) (Tanimoto and Seeliger, 2018).

### Sperm incubation media and sperm preparation

We used Toyoda Yokoyama Hoshi (TYH) media (Cocquet et al., 2010) (138 mM NaCl, 4.8 mM KCl, 2 mM CaCl_2_, 1.2 mM KH_2_PO_4_, 1.0 mM MgSO_4_, 5.6 mM Glucose, 0.5 mM Sodium Pyruvate, 10 mM L-Lactat, 10 mM Hepes) adjusted to pH 7.4 and equilibrated to an osmolality of 290 mosmol^−1^. The cauda epididymus was removed, placed in one well of a four-well multi-dish (Thermo Fisher Scientific) and minced in 500 µl of TYH media. Sperm were allowed to swim out for 15 min. After removing epididymal tissue the sperm suspension was transferred into a 2 ml cup and kept in a 37°C incubator for further analysis.

### Analysis of sperm total motility

Sperm cell motility was analyzed using the computer-assisted sperm analysis (CASAII) system (HT CASA Ceros II, Hamilton Thorne, Beverly, USA) as per guidelines of the supplier. Briefly, sperm cell suspensions from CD-1 and B6/J +/N and N/N and the corresponding littermate control mice (+/+) were diluted three- to four-fold in TYH media before loading 3 µl on a counting chamber slide to obtain quantitative parameters of sperm motility. Three measurements were performed from each sperm suspension. Image collection and quantification of sperm function parameters were achieved with systems default settings. Total sperm cell motility was calculated as a percentage by subtracting the static sperm fraction from the total number of cells.

### Immunoprecipitation

Testis tissue was homogenized in 750 μl lysis buffer (50 mM Tris pH 7.5, 1 mM EDTA, 150 mM NaCl, 1% Triton-X, 1 mM Na3VO4, 5 mM NaF, 1 mM PMSF, 1×Protease inhibitor cocktail). Protein extracts were sheared on ice with 15 1-s pulses at 30% amplitude using a Vibra-Cell sonicator (Sonics) and incubated for 30 min on a rotating wheel at 4°C. After centrifugation at 16,000 ***g*** for 15 min, the supernatant was incubated with 5 μl of anti-ubiquitin ON at 4°C. Immune complexes were precipitated with protein G-sepharose (Pierce, Thermo Fisher Scientific) for 4 h at 4°C and the bead pellets were washed five times with ice-cold lysis buffer. 1×Lämmli was added to the pellets and heated for 5 min at 92°C. After centrifugation the supernatant was subjected to SDS-page.

### *In vitro* fertilization

The IVF protocol is based on methods developed by Naomi Nakagata's laboratory and Infrafontier GmbH (Guan et al., 2014). Female CD-1 mice were superovulated by intraperitoneal injection of 5 IU PMSG (Intervet Deutschland GmbH) followed by 5 IU hCG (Intervet Deutschland GmbH) 50 h later. Mice were euthanized by cervical dislocation 12 h post injection and cumulus-oocyte complexes (COCs) were collected from the oviducts. Cumulus cells were then removed by treating COCs with 300 µg/ml hyaluronidase (Sigma-Aldrich). Oocytes were washed in M2 media (Sigma-Aldrich), transferred to plates (∼30 oocytes in each drop) containing 200 µl HTF media (Quinn et al., 1985) (102 mM NaCl, 4.7 mM KCl, 0.2 mM MgSO_4_, 0.37 mM KH_2_PO_4_, 2.0 mM CaCl_2_, 25 mM NaHCO_3_, 2.8 mM Glucose, 0.33 mM Sodium pyruvate, 21.4 mM Sodium lactate, 0.075 g/l Penicillin G*potassium salt, 0.05 g/l Streptomycin sulfate, 4.0 g/l BSA), covered by mineral oil (Labotect GmbH, Rosdorf, Germany) and incubated until insemination. Sperm were incubated (37°C, 5% CO_2_) in HTF media under mineral oil for 15–20 min to initiate capacitation. Next, 10 µl of sperm suspension was added to the insemination plates and incubated for 4–5 h at 37°C, 5% CO_2_. After fertilization, oocytes were washed 5×in HTF media and transferred for final development into KSOM^AA^ media (95 mM NaCl, 2.5 mM KCl, 0.35 mM KH_2_PO_4_, 0.2 mM MgSO4, 0.2 mM Glucose, 0.06 g/l Penicillin G*potassium salt, 0.05 g/l Streptomycin sulfate, 10 mM Sodium lactate, 25 mM NaHCO_3_, 0.001 g/l Phenol Red, 0.2 mM Sodium pyruvate, 1.7 mM CaCl_2_, 0.01 mM Na_2_-EDTA, 1 mM L-Glutamine, 1.0 g/l BSA, add 0.5 ml of 100× MEM nonessential amino acid and 1 ml of 50× MEM essential amino acid/100 ml KSOM) at 37°C under 5% CO_2_ ON (Ho et al., 1995). 24 h after initiation of insemination two-cell embryos and unfertilized oocytes were counted and imaged on a Leica M60 stereo microscope (Leica Mikrosystem, Wetzlar, Germany).

### Mating and fertility

Thirty CD-1 females were mated with CD-1 +/+ males to assess the capacity to give birth. Reproductive capacity for Best1^Y227N^ and Best1^−/−^ males was assayed by breeding 7 CD-1 N/N and 8 −/− males with two tested +/+ CD-1 females, respectively. After 19 days, females were isolated and kept in holding cages. The total number of litters and pups during the entire mating period was counted.

### Statistical analysis

For comparison between two groups, statistical analysis was performed by two-paired Student's *t*-test. For comparisons among three or more groups, we used one-way analysis of variance (ANOVA). *P*<0.05 was considered statistically significant.

## Supplementary Material

Supplementary information
