# Supplementary material for: The Y227N mutation affects bestrophin-1 protein stability and impairs sperm function in a mouse model of Best vitelliform macular dystrophy
Source: Biol Open. 2019 Jun 14;8(7):bio041335. doi: 10.1242/bio.041335 (PMC6679414; doi:10.1242/bio.041335)
Supplement: Supplementary information [file biolopen-8-041335-s1.pdf]

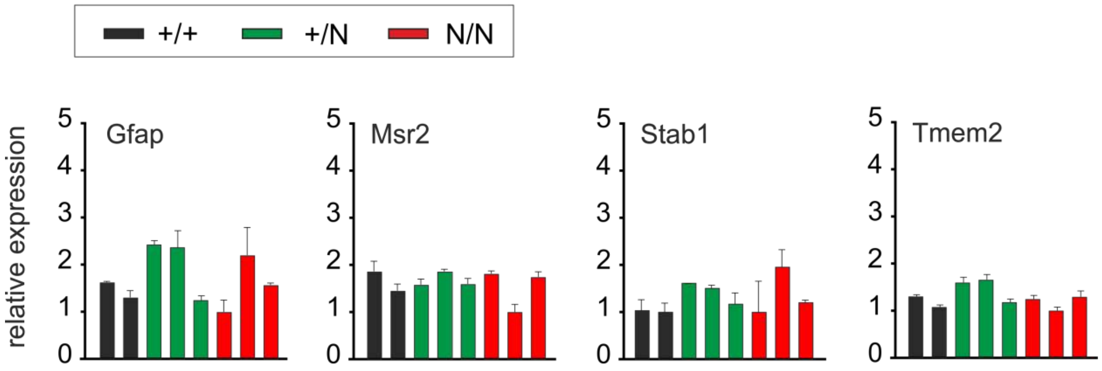

Fig. S1. Effect of mutant Best1 on mRNA expression of genes involved in apoptosis and immune responses. Quantitative real-time RT-PCR expression analysis of Gfap (Glial fibrillary acidic protein), Msr2 (Macrophage scavenger receptor 2), Stab1 (Stabilin 1) and Tmem2 (Transmembrane protein 2) revealing no statistical difference in mRNA expression between eyes from 13 months old B6/J +/+, +/N and N/N mice. Expression was normalized to Hprt1, respectively. RNA was extracted from the RPE/retina complex isolated from eyes of two B6/J +/+ and three +/N and N/N mice, respectively (12 to 13 months of age). The mean ±SD is given for each experiment which was performed in triplicates.

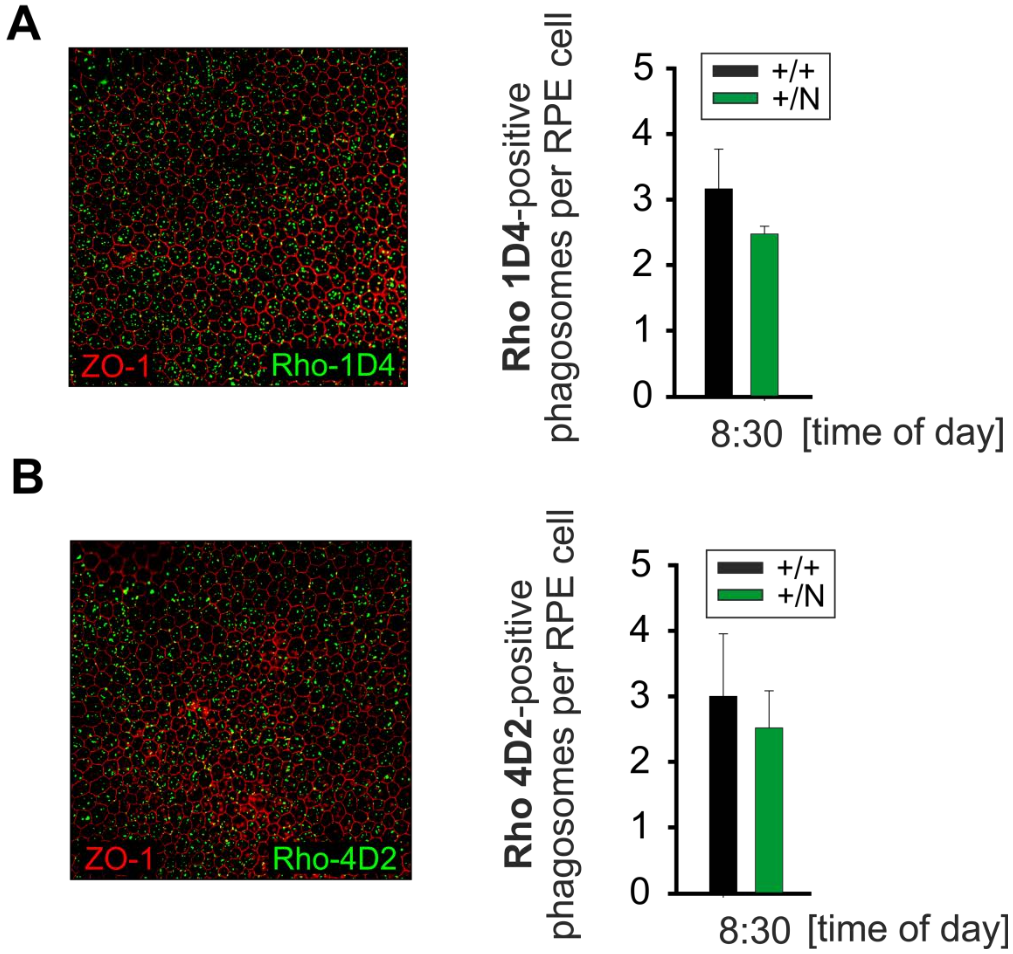

Fig. S2. Comparison of antibodies directed to the N- (Rho-4D2) and C- (Rho-1D4) terminus of rhodopsin. Representative RPE flat mounts from a 6 months old B6/J +/+ mouse 2.5 h after light onset at 8:30am from the left (**A**) and right (**B**) eye. POS were immunostained (green) in RPE flat mounts with C-terminal Rho-1D4 (left eye) and N-terminal Rho-4D2 (right eye) antibodies. Cell boundaries were determined by red staining with ZO-1. The mean of 1D4- and 4D2-positive phagosomes per RPE cell is given from six to eight photographic fields of immunostained flat mounts. The mean ±SD is given for each time point; n = 2 eyes from 2 mice for each antibody.

Table S1. Primer pairs and Roche library probes for quantitative real-time PCR

Gene	F-Primer (5`-3`)	R-Primer (5`-3`)	Roche Library Probe
Mfge8	gtgccctgtgggctactc	gtattggggacggctgtg	58
Itgav	ggtgtggatcgagctgtctt	caaggccagcatttacagtg	21
Intgb5	tttgccaagttccaaagtga	tctgtacagggggtttgagg	1
CD36	ttgaaaagtctcggacattgag	tcagatccgaacacagcgta	6
CD81	tgctcttcgtcttcaatttcg	tgacgcaaccacagagctac	20
Gas6	ggatttgctacctacaggctca	ttaacttcccaggtggtttcc	42
Ptk2	agggtctgatgaagcaccac	actggatctcgggctaggat	3
Mertk	gatggttctggccccact	ctgatctagctcggtctcttcc	45
Pros1	tgtgacaacactcctggaagtta	agcgcattcatccaaatctt	68
Ctsd	gcgtcttgctgctcattct	acttgcgcagagggattct	79
Clta	gagaacgatgaggccttcg	ccattgctctcctggtagtattc	89
Cltb	ttgagaagaacaagatcaacaacag	gggctaccttctcccactct	63
Cltc	cagagacacagcccattgttt	cactgggtcctgctgttagc	45
Hprt1	tcctcctcagaccgctttt	cctggttcatcatcgctaatc	95
Anxa1	ctttgccaagccatcctg	tgggatgtctagtttccacca	21
Ccl2	catccacgtgttggctca	gatcatcttgctggtgaatgagt	62
Ccl6	ccttgtggctgtccttgg	gcgacgatcttctttttcca	64
CD68	ctctctaaggctacaggctgct	tcacggttgcaagagaaaca	27
Casp1	cccactgctgatagggtgac	gcataggtacataagaatgaactgga	103
Casp8	tgaacaatgagatccccaaat	caaaaatttcaagcaggctca	11
Msr2	gcctttgattgtggacatga	gatcttcagaaagtgctgggtaa	67
Tmem2	ctggccatggagaggagat	cgtctaaccttaactttccgttct	82
Gfap	acagactttctccaacctccag	ccttctgacacggatttggt	64
Stab1	gctgatgctctctgcagctat	agcgaagcccagcttacac	81

Biology Open • Supplementary information
